# Comparison of Wait Times for New Patients Between the Private Sector and United States Department of Veterans Affairs Medical Centers

**DOI:** 10.1001/jamanetworkopen.2018.7096

**Published:** 2019-01-18

**Authors:** Madeline Penn, Saurabha Bhatnagar, SreyRam Kuy, Steven Lieberman, Shereef Elnahal, Carolyn Clancy, David Shulkin

**Affiliations:** 1 Office of the Secretary, Department of Veterans Affairs, Washington, DC; 2 Office of Quality, Safety, and Value, Department of Veterans Affairs, Washington, DC; 3 Surgical Service, The Center for Innovations in Quality, Safety, Overton Brooks Veterans Affairs Medical Center, Shreveport, Louisiana; 4 Department of Surgery, Louisiana State University, Shreveport; 5 Acting Principal Deputy Under Secretary for Health, Veterans Health Administration, Department of Veterans Affairs, Washington, DC; 6 New Jersey Department of Health, Trenton; 7 Deputy Under Secretary for Discovery, Education and Affiliate Networks, Veterans Health Administration, Department of Veterans Affairs; 8 United States Department of Veterans Affairs, Gladwyne, Pennsylvania

## Abstract

**Importance:**

Concerns have been raised about the adequacy of health care access among patients cared for within the United States Department of Veterans Affairs (VA) health care system.

**Objectives:**

To determine wait times for new patients receiving care at VA medical centers and compare wait times in the VA medical centers with wait times in the private sector (PS).

**Design, Setting, and Participants:**

A retrospective, repeated cross-sectional study was conducted of new appointment wait times for primary care, dermatology, cardiology, or orthopedics at VA medical centers in 15 major metropolitan areas in 2014 and 2017. Comparison data from the PS came from a published survey that used a secret shopper survey approach. Secondary analyses evaluated the change in overall and unique patients seen in the entire VA system and patient satisfaction survey measures of care access between 2014 and 2017.

**Main Outcomes and Measures:**

The outcome of interest was patient wait time. Wait times in the VA were determined directly from patient scheduling. Wait times in the PS were as reported in Merritt Hawkins surveys using the secret shopper method.

**Results:**

Compared with the PS, overall mean VA wait times for new appointments in 2014 were similar (mean [SD] wait time, 18.7 [7.9] days PS vs 22.5 [7.3] days VA; *P* = .20). Department of Veterans Affairs wait times in 2014 were similar to those in the PS across specialties and regions. In 2017, overall wait times for new appointments in the VA were shorter than in the PS (mean [SD], 17.7 [5.9] vs 29.8 [16.6] days; *P* < .001). This was true in primary care (mean [SD], 20.0 [10.4] vs 40.7 [35.0] days; *P* = .005), dermatology (mean [SD], 15.6 [12.2] vs 32.6 [16.5] days; *P* < .001), and cardiology (mean [SD], 15.3 [12.6] vs 22.8 [10.1] days; *P* = .04). Wait times for orthopedics remained longer in the VA than the PS (mean [SD], 20.9 [13.3] vs 12.4 [5.5] days; *P* = .01), although wait time improved significantly between 2014 and 2017 in the VA for orthopedics while wait times in the PS did not change (change in mean wait times, increased 1.5 days vs decreased 5.4 days; *P* = .02). Secondary analysis demonstrated an increase in the number of unique patients seen and appointment encounters in the VA between 2014 and 2017 (4 996 564 to 5 118 446, and 16 476 461 to 17 331 538, respectively), and patient satisfaction measures of access also improved (satisfaction scores increased by 1.4%, 3.0%, and 4.0% for specialty care, routine primary care, and urgent primary care, *P* < .05).

**Conclusions and Relevance:**

Although wait times in the VA and PS appeared to be similar in 2014, there have been interval improvements in VA wait times since then, while wait times in the PS appear to be static. These findings suggest that access to care within the VA has improved over time.

## Key Points

**Question:**

How do wait times for outpatient appointments compare between United States Department of Veterans Affairs (VA) and private sector hospitals?

**Findings:**

In this repeated cross-sectional study of wait time data from VA facilities and private sector hospitals in primary care, dermatology, cardiology, and orthopedics from 15 major metropolitan areas, there was no statistically significant difference between private sector and VA mean wait times in 2014. In 2017, mean wait times were statistically significantly shorter for the VA compared with the private sector facilities as wait times from 2014 to 2017 improved in the VA facilities while wait times in the private sector remained unchanged.

**Meaning:**

Access to care within VA facilities appears to have improved between 2014 and 2017 and appears to have surpassed access in the private sector for 3 of the 4 specialties evaluated.

## Teaser

This cross-sectional study examines appointment wait times in the private sector and Veterans Affairs health care system to determine their differences and changes over time.

## Introduction

In 2014, reports indicated that veterans were waiting too long for care and that scheduling data may have been manipulated at a United States Department of Veterans Affairs (VA) facility in Phoenix, Arizona.^1^ This incident damaged the VA’s credibility and created a public perception regarding the VA health care system’s inability to see patients in a timely manner.^2^ In response, the VA has worked to improve access, including primary care, mental health, and other specialty care services.^3^ There is evidence suggesting that these efforts have improved access to care, including reports that 22% of VA patients are now seen on the same day as the requested appointment.^4^ Despite these efforts, the adequacy of access to VA care remains unclear.

Although prior studies have offered potential options for defining optimal wait times, to date, there are no established benchmarks for a reasonable wait time for new patients requiring primary or specialty care.^5^ Reviews of the available evidence on care timeliness suggest that wait time data are limited and poorly understood.^6^ In hopes of improving insights on care timeliness, the VA tracks and publicly reports on wait times.^7^ However, formal analyses of these data are limited and comparisons with private sector (PS) wait times have not been reported to date.

Merritt Hawkins (MH) has used survey methods to publish PS wait times for primary and specialty care.^8^ These survey data afford an opportunity to compare wait times in the VA with those in the PS and assess for temporal changes in wait times in these settings. Accordingly, we performed a repeated cross-sectional analysis of wait times in 2014 and 2017 as determined from VA scheduling data for primary care, dermatology, cardiology, and orthopedics in metropolitan areas. Wait times in the VA were then compared with PS wait times as determined from published survey data.

## Methods

### Data Source and Study Population

#### VA Wait Time Data and Study Population

We identified all patients requesting a new-care appointment within the VA for primary care, dermatology, cardiology, or orthopedics in 2014 and 2017. These specialties and years were selected to facilitate comparison with MH survey data. Although the MH survey also includes data on obstetrics and gynecology wait times, the VA collects data for comprehensive women’s health and gynecology only, as the VA does not directly provide obstetrical care. We then restricted our analysis to patients seeking care within a 50-mile radius of the global positioning system center of a city that had both VA data on wait times for the subspecialty of interest and comparison data in MH survey data. Wait times were calculated by counting the number of days between the day that a veteran requested an appointment to the date of the appointment. For example, if a patient were to call and schedule an appointment on a Wednesday and the appointment is then scheduled for the following Tuesday, the wait time is computed to 6 days. This wait time may not always be the earliest available appointment. If a veteran is unavailable or declines the initial earliest available appointment and asks to be seen on another date, the wait time is measured to this later, scheduled appointment. The VA data were analyzed directly from the VA medical centers scheduling system and did not involve surveying veterans or clinicians. Wait times measured this way at the VA medical centers are calculated automatically based on the request and appointment date and cannot be modified by staff. All VA data are deidentified, publicly available, and in aggregate form. In accordance with VA institutional review board, this study, involving published and deidentified information, is exempted; thus, no institutional review board approval was needed for this information. This study followed the Strengthening the Reporting of Observational Studies in Epidemiology (STROBE) reporting guideline.

#### Merritt Hawkins

Merritt Hawkins published a survey examining wait times from the data of new-patient appointment requests for 15 major metropolitan areas in 2014 and in both 15 major and 15 midsized metropolitan areas (sized between 88 000 and 143 000 people) in 2017.^8^ Wait times were determined using the secret shopper method.^9^ Secret shopper is an approach where research associates called physician offices in the 30 metropolitan areas to schedule a new patient appointment. Research associates used a verbal script and inquired about the first available time for a new-patient appointment. The MH script was intended to yield the earliest available appointment. Merritt Hawkins collected data for 5 specialties: cardiology, dermatology, family medicine, orthopedics, and obstetrics/gynecology. The research associates contacted a minimum of 10 separate physician offices per specialty per large metropolitan area (with 20 being the preferred goal) and a minimum of 5 separate physician offices per specialty per midsized metropolitan area (with 10 being the preferred goal). In large metropolitan markets, 1414 medical offices were surveyed and, in midsized metropolitan markets, 494 medical offices were surveyed.

To make the resulting sample representative of the catchment area, physician offices were randomly selected from internet-based physician office listings, such as the online yellow pages, a preferred provider organization physician directory, or Healthgrades. The Merritt Hawkins method was intended to replicate the experience of a patient new to a community seeking to schedule a nonemergent physician appointment. The mean wait times for each specialty in each metropolitan region were reported in aggregate form.

### Primary Outcome Measure

The outcome of interest was patient wait time. Wait times in the VA were determined directly from patient scheduling as described above. Wait times in the PS were as reported in MH surveys using the secret shopper method as described above.

### Statistical Analysis

We used linear regression to compare mean new-appointment wait times between the VA and PS. The primary analyses included data on all metropolitan areas and specialties surveyed by MH with comparators within the VA. Comparisons were made for overall wait times in 2014 and 2017, and change in wait times between 2014 and 2017. We then repeated these analyses stratified by specialty and metropolitan area. In the comparison of metropolitan areas, we also performed a 2-tailed sign test to analyze the significance of the number of regions that had shorter wait times for the comparisons conducted. All regression analyses were unadjusted and were performed using Excel, version 1708 (Microsoft Corp), with statistical significance judged at the *P* = .05 level. To evaluate trends in responses to Consumer Assessment of Healthcare Providers and Systems (CAHPS)^10^ regarding a patient’s experience obtaining care, we conducted a 2-tailed, Mann-Kendall trend test, with statistical significance judged at the *P* = .05 level to test the null hypothesis that no monotonic trend is present.

### VA Patient Volumes and Patient Experience in Obtaining Care

As a secondary analysis to further explore for evidence of changing outpatient access to VA care, we evaluated trends in the volume of patients seen at VA facilities for primary care, dermatology, cardiology, and orthopedics as determined from VA data on unique patients seen and appointment encounters published by the National Center for Veterans Analysis and Statistics.^11^

We also evaluated VA-collected CAHPS data and VA-selected items from Clinician Group CAHPS 3.0 that inquire about a patient’s experience obtaining care needed for either routine problems or needed right away. The VA uses Clinician and Group CAHPS 3.0 to evaluate both primary care and specialty care. Patients were asked whether they always, usually, sometimes, rarely, or never got an appointment right away. In the CAHPS, top box responses indicate that the patients stated they always got an appointment right away and top 2 box responses indicated that the patients stated they always or usually got an appointment right away. Although we lacked PS comparator data, prior studies have suggested using these survey metrics as the optimal approach to evaluate access in health systems.^12^

## Results

### Overall VA and PS Comparison

**Figure 1.**
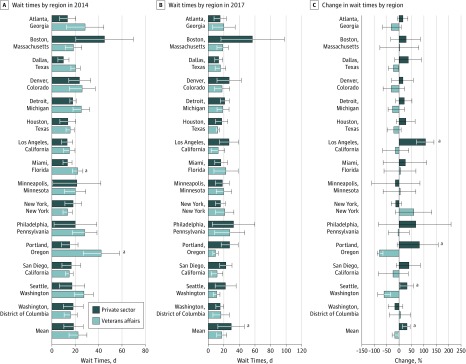
Mean Wait Times and Changes by Region for Private Sector and Veterans Affairs Hospitals, Stratified by Region A, Wait time in 2014. B, Wait time in 2017. C, Percent change in wait times between 2014 and 2017. Percentages greater than 0 indicate increased wait time; negative percentages indicate decreased wait time. Error bars indicate SD for each private sector and Veterans Affairs region. ^a^Significant comparison at *P* = .05.

In 2014, there was no statistically significant difference between VA and PS mean (SD) wait times (22.5 [7.3] vs 18.7 [7.9] days; *P* = .20) (Figure 1A; eTable 1 in the Supplement). In 2017, the VA had a mean wait time that was 12 days shorter than wait times in the PS (mean [SD], 17.7 [5.9] vs 29.8 [16.6] days; *P* < .001) (Figure 1B; eTable 2 in the Supplement).

The mean VA wait times improved by 4.92 days from 2014 to 2017 (from mean [SD] of 22.5 [7.3] to 17.6 [4.9] days; *P* = .046) (Figure 1C; eTable 3 in the Supplement). In contrast, PS wait times had no statistically significant change in wait times between 2014 and 2017.

### Wait Times by Specialty

In 2014, there was no statistically significant difference between PS and VA wait times for cardiology, primary care, and dermatology (eTable 1 in the Supplement). However, VA facilities had statistically significantly longer wait times for orthopedics compared with the PS (mean [SD], 9.9 [4.7] vs 23.9 [8.1] days; *P* < .001).

**Figure 2.**
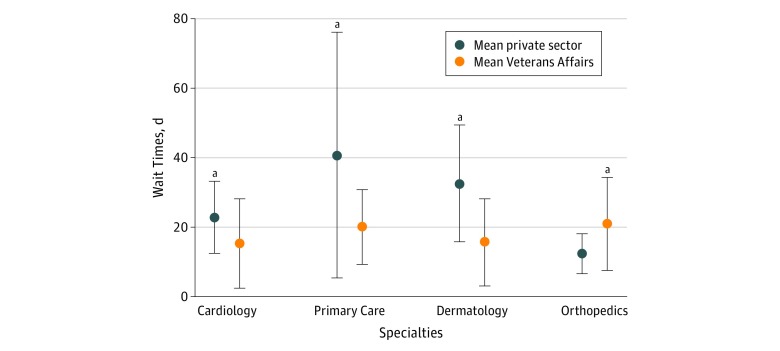
Wait Time by Specialty Each dot represents the mean wait time per new patient for an appointment in the private sector and Veterans Affairs facilities in 2017, stratified by specialty of care. The error bars indicate measures of uncertainty in SD. ^a^Significant comparison at *P* = .05.

In 2017, VA facilities had statistically significantly shorter mean wait times for 3 of the 4 analyzed specialties: cardiology (mean [SD], 15.3 [12.6] vs 22.8 [10.1] days; *P* = .04), primary care (mean [SD], 20.0 [10.4] vs 40.7 [35.0] days; *P* = .005), and dermatology (mean [SD], 15.6 [12.2] vs 32.6 [16.5] days; *P* < .001) (Figure 2). The VA facilities had statistically significantly longer wait times for orthopedics (mean [SD], 20.9 [13.3] vs 12.4 [5.5] days; *P* = .01).

For both the VA and PS, there was no significant change to mean wait times by specialty for cardiology, primary care, and dermatology between 2014 and 2017. However, PS wait times increased in 12 of the 15 regions for orthopedics (80%; sign test *P* = .01), with no significant change to mean wait time (9.9 vs 11.4 days; *P* = .33). In contrast, mean VA wait times for orthopedics decreased by 5.4 days between 2014 and 2017 (from 23.9 to 18.5 days; *P* = .05).

### Wait Times by Geographic Area

In 2014, the PS had statistically significantly shorter mean wait times in Dallas, Texas (10.3 vs 20.6 days; *P* = .02), Miami, Florida (13.8 vs 22.3 days; *P* = .03), and Portland, Oregon (15.5 vs 42.3 days; *P* = .047), and wait times similar to those of the VA for the other 12 regions. The overall number of metropolitan areas for which the PS had shorter wait times than the VA for outpatient services was not significant by the sign test (sign test *P* = .30).

In 2017, there were no metropolitan areas with statistically significant differences in wait times between the PS and VA. However, the point estimate mean wait time was shorter in the VA compared with the PS for 22 of the 30 metropolitan areas (73.3%; sign test *P* = .02).

Although VA mean wait times improved for most regions (73.3% of the 15 major regions analyzed), the number of metropolitan areas for which wait times improved was statistically nonsignificant (improved in 11 of 15 regions; sign test *P* = .12). In contrast, the PS had increasing wait times in 12 of the 15 metropolitan areas (80%; sign test *P* = .04).

### Secondary Analysis

**Figure 3.**
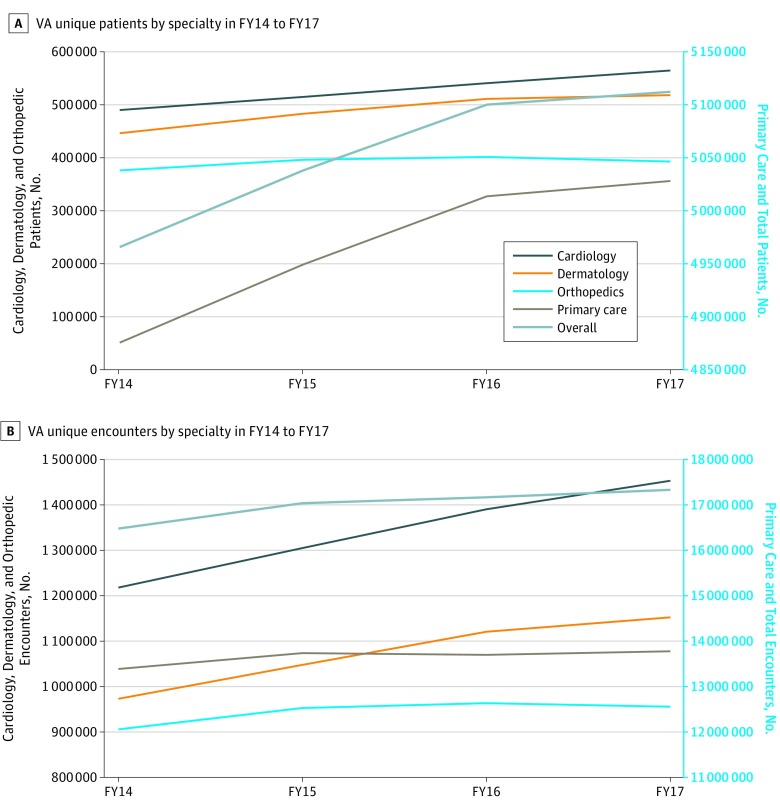
Number of Unique Patients and Encounters Within the Veterans Affairs (VA) System A, The number of unique patients enrolled in the VA annually by specialty overall. B, The number of unique encounters in the VA annually by specialty and overall. FY indicates fiscal year.

Within unique VA patients, 9.4% are women. In addition, of all unique veteran patients, 1.4% are younger than 24 years, 18.5% are between 25 and 44 years, 33.0% are between 45 and 64 years, 32.4% are between 65 and 79 years, and 14.7% are 80 years or older. The overall number of unique patients seen and the volume of encounters nationally increased between fiscal year 2014 (FY14) and FY17 (FY starting in October) from 4 996 564 (FY14) to 5 118 446 (FY17) unique patients and 16 476 461 (FY14) to 17 331 538 (FY17) unique encounters (Figure 3). The number of unique patients and volume of encounters increased for all specialties.

We observed statistically significant annual incremental improvements in VA CAHPS scores for both urgent and routine primary care and specialty care appointments (Table). For urgent primary care, “I always got an appointment for care needed right away” improved by a mean of 1.3% annually and 4.0% cumulatively (from 44.0% of patients in FY14 to 48.0% of patients FY17; *P* = .04). For routine primary care, “I always got an appointment for routine care” improved by a mean of 1.0% annually and 3.0% cumulatively (from 53.0% of patients in FY14 to 56.0% of patients FY17; *P* = .048). For urgent specialty care, “I always got an appointment for care needed right away” improved 1.4% (from 44.7% of patients in FY16 to 46.1% of patients FY17; *P* = .01). For routine specialty care, “I always got an appointment for routine care” improved 1.4% (from 52.2% of patients in FY16 to 53.6% of patients FY17; *P* = .01).

**Table.** VA Consumer Assessment of Health Care Professionals and Systems Data From FY13 to FY17 in Primary and Specialty Care for Urgent and Routine Appointments

Type of Care	Response^a^	FY, No. (%)					*P* Value^b^
		2013	2014	2015	2016	2017	
**Primary Care**							
Urgent	Top box	47 903 (46.0)	45 441 (44.0)	48 307 (43.6)	48 719 (46.9)	47 664 (48.0)	.04
	Top 2 boxes	75 500 (72.5)	72 293 (70.0)	76 782 (69.3)	75 312 (72.5)	73 681 (74.2)	.05
	Total No.	104 138	103 276	110 796	103 879	99 300	NA
Routine care	Top box	110 949 (54.6)	106 809 (53.0)	115 303 (52.4)	119 190 (55.4)	115 904 (56.0)	.048
	Top 2 boxes	168 862 (83.1)	164 245 (81.5)	178 676 (81.2)	179 860 (83.6)	174 891 (84.5)	.04
	Total No.	203 203	201 527	220 044	215 144	206 972	NA
**Specialty Care**							
Urgent	Top box	NA	NA	NA	40 096 (44.7)	38 768 (46.1)	.01
	Top 2 boxes	NA	NA	NA	64 943 (72.4)	62 314 (74.1)	.008
	Total No.	NA	NA	NA	89 700	84 095	NA
Routine care	Top box	NA	NA	NA	87 783 (52.2)	86 722 (53.6)	.01
	Top 2 boxes	NA	NA	NA	138 232 (82.2)	135 098 (83.5)	<.001
	Total No.	NA	NA	NA	168 166	161 794	NA

Abbreviations: FY, fiscal year; NA, not applicable; VA, Veterans Affairs.

^a^ In urgent care, the top box response means that the individual surveyed stated “always got appointment for care needed right away”; the top 2 boxes response means that the individual surveyed stated either “always got appointment for care needed right away” or “usually got appointment for care needed right away.” In routine care, the top box response means that the individual surveyed stated “always got appointment for routine care”; the top 2 boxes response means that the individual surveyed stated either “always got appointment for routine care” or “usually got appointment for routine care.”

^b^ *P* values were calculated using a 2-tailed Mann-Kendall test. *P* values less than the significant level (α = .05) reject the null hypothesis, indicating a significantly increasing trend line for Consumer Assessment of Healthcare Providers and Systems scores.

## Discussion

In a comparison of VA wait times for new appointments determined from the VA scheduling system with a market survey of new-appointment wait times for the PS, the VA had similar new appointment wait times to the PS in FY2014. Overall new-appointment wait times in the VA were shorter than in the PS in FY2017 and for the specialties of primary care, cardiology, and dermatology. The mean FY2017 PS wait times for primary care and dermatology were more than double the VA mean wait times. In FY2017, wait times for orthopedics remained longer in the VA compared with the PS, although wait times improved overall and for orthopedics in the VA between FY2014 and FY2017, while wait times in the PS remained static and PS orthopedic wait times increased. Concurrently, there was an increase in the number of unique patients seen, volume of encounters, and an improvement in CAHPS access score ratings within the VA, further supporting the finding that access to care has improved over time within the VA.

Outpatient access is an important issue. The association between wait times for a first appointment and patient outcomes is especially important for elderly and vulnerable populations because delayed access to health care is associated with poor health.^13^ Congress commissioned the Veterans Choice Act Independent Assessment that analyzed VA wait times and provided recommendations on how to improve them.^14^ To our knowledge, this analysis is the first to compare VA and PS wait times. Our findings suggest that wait times in the PS have remained static and wait times have improved in the VA. As a result, VA wait times in FY2017 were shorter than in the PS for primary care, dermatology, and cardiology. Improvements in VA wait times for orthopedics have closed the gap, although wait times in the PS remain shorter for this specialty.

With data from both PS and VA facilities, further analyses of wait times can be conducted to identify needed change in access. Because of the comparison of the PS with the VA, the results of this study suggest that VA initiatives, rather than changes to the overall health services market, led to the decrease in wait times, increase in the unique number of patients seen and volume of encounters, and improvement in CAHPS access score ratings within the VA.

### Limitations

This study has limitations. The method for collecting wait times was different between the MH report and VA data. However, the MH survey method may lead to reporting shorter wait times for the PS. For the secret shoppers method, the research associates at MH called physicians’ offices asking to be told the first available time for a new-patient appointment. This earliest availability was recorded as the wait time. However, the VA data record scheduled wait times, which may not reflect the earliest available appointment. If a veteran declines the initial appointment and asks to be seen later, this delayed scheduled appointment is the wait time entered and documented. In addition, if the distributions within specialty and region are skewed, the median will be a better measure of centrality than the mean; however, because the MH survey data reported only arithmetic means for each specialty and region, for consistency, the comparison was made between means of the PS and VA.

Although an evaluation of mental health wait times is important, this study did not include these wait times because the MH survey data did not evaluate them in the PS. In addition, we were unable to compare VA data for rural areas or for midsized areas in FY2014 with those of the PS since MH did not publish these wait times. In line with the data provided by MH, this analysis excluded rural markets and follow-up care. Although this study was a comparative analysis of 30 large metropolitan regions in the United States, follow-up studies are critical to analyze access to the entirety of VA health care with the absence given that nearly one-quarter of veterans live in rural areas.^15^ In addition, the MH data were not independently validated and have limitations; however, since there are no other good, available PS comparisons, MH provided the best usable data set.

## Conclusions

Our study supports the premise that VA wait times have improved between FY2014 and FY2017 and that VA facilities have shorter wait times than the PS across a range of specialties. An analysis of access to mental health services and of access in rural areas in the VA and PS would be useful to pursue in further research. Although the results reflect positively on the VA, we intend to continue improving wait times, the accuracy of the data captured, and the transparency of reporting information to veterans and the public.

## Notes

Supplement. **eTable 1.** Mean Wait Times for Private Sector and Veterans Affairs by Region and Specialty in 2014 **eTable 2.** Mean Wait Times for Private Sector and Veterans Affairs by Region and Specialty in 2017 **eTable 3.** Change in Mean Wait Times from 2014 to 2017 for Private Sector and Veterans Affairs Facilities
