# Supplementary material for: Comparison of Wait Times for New Patients Between the Private Sector and United States Department of Veterans Affairs Medical Centers
Source: JAMA Netw Open. 2019 Jan 18;2(1):e187096. doi: 10.1001/jamanetworkopen.2018.7096 (PMC6484544; doi:10.1001/jamanetworkopen.2018.7096)
Supplement: Supplement. — eTable 1. Mean Wait Times for Private Sector and Veterans Affairs by Region and Specialty in 2014 eTable 2. Mean Wait Times for Private Sector and Veterans Affairs by Region and Specialty in 2017 eTable 3. Change in Mean Wait Times from 2014 to 2017 for Private Sector and Veterans Affairs Facilities [file jamanetwopen-2-e187096-s001.pdf]

Supplementary Online Content

Penn M, Bhatnagar S, Kuy S, et al. Comparison of wait times for new patients between the private sector and United States Department of Veterans Affairs medical centers. *JAMA Netw Open*. 2019;2(1):e187096. doi:10.1001/jamanetworkopen.2018.7096

eTable 1. Mean Wait Times for Private Sector and Veterans Affairs by Region and Specialty in 2014

eTable 2. Mean Wait Times for Private Sector and Veterans Affairs by Region and Specialty in 2017

eTable 3. Change in Mean Wait Times from 2014 to 2017 for Private Sector and Veterans Affairs Facilities

This supplementary material has been provided by the authors to give readers additional information about their work.

© 2019 Penn M et al. *JAMA Network Open.*

eTable 1. Mean Wait Times for Private Sector and Veterans Affairs by Region and Specialty in 2014.

City	Cardiology		Family Care/Primary Care		Dermatology		Orthopedics		Mean (SD)	
	PS[1]	VA[2]	PS	VA	PS	VA	PS	VA	PS	VS
Atlanta, Georgia	11	39	24	41	14	2	6	33	14 (7)	29 (16)
Boston, Massachusetts	27	25	66	25	72	16	16	9	45 (24)	19 (7)
Dallas, Texas	11	19	5	23	17	25	8	15	10 (4)	21 (4)
Denver, Colorado	28	40	16	27	37	10	15	27	24 (9)	26 (11)
Detroit, Michigan	17	23	16	16	22	32	18	32	18 (2)	26 (7)
Houston, Texas	11	13	19	19	21	19	5	12	14 (6)	16 (3)
Los Angeles, California	12	8	20	19	14	15	7	19	13 (5)	15 (4)
Miami, Florida	18	16	12	26	16	21	9	26	14 (3)	22 (4)
Minneapolis, Minnesota	15	7	10	18	56	27	5	29	22 (20)	20 (9)
New York, New York	15	11	26	14	24	11	9	20	19 (7)	14 (4)
Philadelphia, Pennsylvania	6	37	21	28	49	12	5	36	20 (18)	28 (10)
Portland, Oregon	12	25	13	55	27	59	10	30	16 (7)	42 (15)
San Diego, California	28	17	7	14	14	11	18	18	17 (8)	15 (3)
Seattle, Washington	9	39	23	19	32	23	6	30	18 (11)	28 (8)
Washington, District of Columbia	32	18	14	19	17	8	11	21	19 (8)	16 (5)

[1] Private Sector

[2] Department of Veterans Affairs

© 2019 Penn M et al. *JAMA Network Open.*

eTable 2. Mean Wait Times for Private Sector and Veterans Affairs by Region and Specialty in 2017[1,2]

	Cardiology		Family Care/ Primary Care		Dermatology		Orthopedics		Mean (SD)	
**City**	**PS,[3] days**	**VA,[4] days**	**PS, days**	**VA, days**	**PS, days**	**VA, days**	**PS, days**	**VA, days**	**PS, days**	**VA, days**
**Mid-Sized Markets**										
Albany, New York	10	5	122	39	46	15	11	8	**47.25 (45.53)**	**16.75 (13.35)**
Billings, Montana	22	7	7	13	11	7	8	67	**12.00 (5.96)**	**23.50 (25.23)**
Cedar Rapids, Iowa	10	21	75	23	91		14		**42.50 (32.50)**	**22.00 (1.00)**
Dayton, Ohio	21	20	40	21	23	20	12	12	**24.00 (10.12)**	**18.25 (3.63)**
Evansville, Indiana	30	19	76	14	43	54	34	16	**45.75 (18.09)**	**25.75 (16.41)**
Fargo, North Dakota	39	19	20	19	71	29	10	19	**35.00 (23.25)**	**21.50 (4.33)**
Fort Smith, Arkansas	48	19	37	16	10	3	23		**31.67 (15.97)**	**12.67 (6.94)**
Hampton, Virginia	16	5	35	15	25	28	13	51	**22.25 (8.58)**	**24.75 (17.21)**
Hartford, Connecticut	40	19	60	48	47	22	15		**49.00 (8.29)**	**29.67 (13.02)**
Lafayette, Louisiana	31	10	10	13	38	4	11		**26.33 (11.90)**	**9.00 (3.74)**
Manchester, New Hampshire	46		72		33	15	14	23	**23.50 (9.50)**	**19.00 (4.00)**
Odessa, Texas	63		24	12	6		17		**24.00 (0)**	**12.00 (0)**
Savannah, Georgia	36		61	23	26	6	14		**43.50 (17.50)**	**14.50 (8.50)**
Temecula, California	55		22	9	25	4	13	8	**20.00 (5.10)**	**7.00 (2.16)**
Yakima, Washington	18		153	18	31	2	16		**92.00 (61.00)**	**10.00 (8.00)**
**Large Markets**										
Atlanta, Georgia	16	42	27	18	13	3	7	19	**15.75 (7.26)**	**20.31 (14.04)**
Boston, Massachusetts	45		109	27	52	11	11	19	**57.33 (40.19)**	**19.07 (6.45)**
Dallas, Texas	12	11	12	16	22	27	10	11	**14.00 (4.69)**	**16.20 (6.45)**
Denver, Colorado	22	14	27	19	51	7	10	33	**27.50 (14.91)**	**18.26 (9.51)**
Detroit, Michigan	14	17	27	13	27	32	19	16	**21.75 (5.54)**	**19.50 (7.37)**
Houston, Texas	12	14	21	12	28	11	10	14	**17.75 (7.22)**	**12.82 (1.37)**
Los Angeles, California	20	2	42	10	35	23	12	19	**27.25 (11.86)**	**13.27 (8.08)**
Miami, Florida	14		28	44	11	9	12	17	**17.00 (7.79)**	**23.27 (15.03)**
Minneapolis, Minnesota	22	15	8	13	30	37	15	18	**18.75 (8.17)**	**20.75 (9.55)**
New York, New York	15	11	26	39	15	12	10	25	**16.50 (5.85)**	**21.73 (11.51)**
Philadelphia, Pennsylvania	28	59	17	26	78	9	10	19	**33.25 (26.62)**	**28.14 (18.87)**
Portland, Oregon	32	6	39	13	30	8	11	13	**28.00 (10.37)**	**10.08 (3.03)**
San Diego, California	30	3	13	8	30	20	19	16	**23.00 (7.31)**	**11.83 (6.70)**
Seattle, Washington	16	5	26	13	42	16	7	11	**22.75 (12.99)**	**11.25 (4.02)**
Washington, District of Columbia	18	10	17	27	20	4	8	27	**15.75 (4.60)**	**17.02 (10.20)**

[1] Averages are calculated using a region/specialty's data only if it is present in both Private Sector and Veterans Affairs samples.
[2] Some data is missing due to lack of reporting at the specific VA facilities.
[3] Private Sector
[4] Department of Veterans Affairs

© 2019 Penn M et al. *JAMA Network Open.*

eTable 3. Change in Mean Wait Times from 2014 to 2017 for Private Sector and Veterans Affairs Facilities.

	Overall Change between 2014 and 2017	
City	Private Sector, wait time change (days 2017-days 2014)	Veterans Affairs, wait time change (days 2017-days 2014)
Atlanta, Georgia	2.0	-8.4
Boston, Massachusetts	9.0	0.4
Dallas, Texas	3.8	-4.4
Denver, Colorado	3.5	-7.9
Detroit, Michigan	3.5	-6.0
Houston, Texas	3.8	-3.1
Los Angeles, California	14.0	-2.0
Miami, Florida	2.5	1.1
Minneapolis, Minnesota	-2.8	0.5
New York, New York	-2.0	8.0
Philadelphia, Pennsylvania	13.0	-0.1
Portland, Oregon	12.5	-32.3
San Diego, California	6.3	-3.4
Seattle, Washington	5.3	-16.5
Washington, District of Columbia	-2.8	0.7
**Mean (SD)**	**4.8 (5.2)**	**-4.9 (9.1)**

© 2019 Penn M et al. *JAMA Network Open.*
